# Influence of Socioeconomic Status on the Association Between Pregnancy Complications and Premature Coronary Artery Disease: Linking Three Cohorts

**DOI:** 10.1089/whr.2023.0092

**Published:** 2024-02-16

**Authors:** Adeel Khoja, Prabha H. Andraweera, Rosanna Tavella, Tiffany K. Gill, Gustaaf A. Dekker, Claire T. Roberts, Suzanne Edwards, Margaret A. Arstall

**Affiliations:** ^1^ Department of Medicine, Adelaide Medical School, Faculty of Health and Medical Sciences, The University of Adelaide, Adelaide, Australia.; ^2^ Department of Medicine, The Robinson Research Institute, The University of Adelaide, Adelaide, Australia.; ^3^ Department of Cardiology, Cardiology Unit, Northern Adelaide Local Health Network, Adelaide, Australia.; ^4^ Department of Cardiology, Basil Hetzel Institute for Translational Health Research, The Queen Elizabeth Hospital, Woodville South, Australia.; ^5^ Department of Obstetrics and Gynaecology, Lyell McEwin Hospital, University of Adelaide, Adelaide, Australia.; ^6^ Department of Medicine, Flinders Health and Medical Research Institute, Flinders University, Bedford Park, Australia.; ^7^ Department of Medicine, Medical Specialties, Faculty of Health Sciences, The University of Adelaide, Adelaide, Australia.

**Keywords:** coronary artery disease, socioeconomic status, pregnancy complications, premature, data linkage, databases

## Abstract

**Background:**

We hypothesized that there is an influence of socioeconomic status (SES) on association between pregnancy complications and premature coronary artery disease (PCAD) risk.

**Materials and Methods:**

This project involved a data linkage approach merging three databases of South Australian cohorts using retrospective, age-matched case–control study design. Cases (*n* = 721), that is, women aged <60 years from Coronary Angiogram Database of South Australia (CADOSA) were linked to South Australian Perinatal Statistics Collection (SAPSC) to ascertain prior pregnancy outcomes and SES. Controls (*n* = 194) were selected from North West Adelaide Health Study (NWAHS), comprising women who were healthy or had health conditions unrelated to CAD, age matched to CADOSA (±5 years), and linked to SAPSC to determine prior pregnancy outcomes and SES. This project performed comparative analysis of SES using socioeconomic indexes for areas–index of relative socioeconomic advantage and disadvantage (SEIFA-IRSAD) scores across three databases.

**Results:**

Findings revealed that SEIFA-IRSAD scores at the time of pregnancy (*p*-value = 0.005) and increase in SEIFA-IRSAD scores over time (*p*-value = 0.040) were significantly associated with PCAD. In addition, when models were adjusted for SEIFA-IRSAD scores at the time of pregnancy and age, risk factors including placenta-mediated pregnancy complications such as preterm birth (odds ratio [OR] = 4.77, 95% confidence interval [CI]: 1.74–13.03) and history of a miscarriage (OR = 2.14, 95% CI: 1.02–4.49), and cardiovascular disease (CVD) risk factors including smoking (OR = 8.60, 95% CI: 3.25–22.75) were significantly associated with PCAD. When the model was adjusted for change in SEIFA-IRSAD scores (from CADOSA/NWAHS to SAPSC) and age, pregnancy-mediated pregnancy complications including preterm birth (OR = 4.40, 95% CI: 1.61–12.05) and history of a miscarriage (OR = 2.09, 95% CI: 1.00–4.35), and CVD risk factor smoking (OR = 8.75, 95% CI: 3.32–23.07) were significantly associated with PCAD.

**Conclusion:**

SES at the time of pregnancy and change in SES were not associated with PCAD risk.

## Introduction

Coronary artery disease (CAD) is most commonly owing to atherosclerotic plaque development in the coronary arteries obstructing blood flow to the myocardium. It is generally agreed that an obstruction of an epicardial coronary artery by >50% is clinically relevant in the absence of demonstrated ischemia or symptoms.^1^ Premature coronary artery disease (PCAD) among women is defined as a diagnosis of CAD before 60 years of age as per the Dutch Lipid Clinic Network Score.^2^

Traditional cardiovascular disease (CVD) risk factors such as family history, smoking, hypertension, obesity, diabetes, and dyslipidemia do not account for the total observed burden of CAD in women.^3,4^ Of interest, a large number of studies have shown that women with a history of pregnancy complications, including preeclampsia, gestational diabetes mellitus (GDM), those who deliver a growth restricted baby, or spontaneously deliver preterm (<37 weeks' gestation) are at twice the risk of developing CAD compared with women who do not experience any of these pregnancy complications.^5–13^ The strongest evidence linking pregnancy complications with CAD comes from studies on preeclampsia that has resulted in it being recognized by the American Heart Association as a female-specific cardiovascular risk factor.^9^ Recurrent miscarriage also portends a doubling of the risk of CAD.^11^

Most studies have investigated the association between pregnancy complications and either morbidity or mortality because of CAD or the association between pregnancy complications and conventional CVD risk factors. In Australia, the above-mentioned pregnancy complications are common and are prevalent in ∼25% of all first pregnancies,^14^ suggesting these complications are an important cardiovascular red flag.

Socioeconomic status (SES) plays an important role in determining perinatal outcomes. In a population-based study conducted in Nova Scotia, Canada, it was revealed that the prevalence of GDM, small for gestational age (SGA) and infant mortality, were higher among women with low family income or low SES compared with high family income or high SES despite having access to universal health care services.^15^ Another study from Korea that used a national level database also indicated similar findings that women in the lower SES group had higher rates of abortion, caesarean delivery, preeclampsia, preterm delivery, and obstetric hemorrhage compared with those in the higher SES group.^16^

SES has a quantifiable and significant impact on cardiovascular health. Women in low SES groups, especially those living in poverty, tend to be disproportionately affected by disparities in education, income, and access to health care resources. This disparity inevitably results in increased risk of ischemic heart disease and rehospitalization rates owing to CVDs compared with women in high SES group.^17,18^ There is compelling evidence that shows an independent association between SES and cardiovascular outcomes (including mortality) that is comparable in strength and consistency with any major CVD risk factor.^19^ In addition, a recent study on American adults using data from National Health and Nutrition Examination Survey demonstrated an inverse association (dose-dependent) of family income with the prevalence of CAD.^20^

The associations between SES and pregnancy complications, and also SES and CVD, are well established. However, the role of SES on the association between pregnancy complications and risk of PCAD has not been explored. To address this gap in knowledge, this study aimed to assess the role of SES on the association between pregnancy complications and CAD risk among women aged <60 years.

## Materials and Methods

### Study design

This data linkage project used a retrospective, age-matched case–control study design by merging three databases of South Australian cohorts: Coronary Angiogram Database of South Australia (CADOSA), South Australian Perinatal Statistics Collection (SAPSC), and North West Adelaide Health Study (NWAHS). This project performed a comparative analysis of SES using socioeconomic indexes for areas–index of relative socioeconomic advantage and disadvantage (SEIFA-IRSAD) scores for the three databases. SEIFA is a product developed by the Australian Bureau of Statistics (ABS) that ranks areas in Australia according to relative socioeconomic advantage and disadvantage.^21^ SEIFA 2016 is the latest version and has four indexes. One of the indexes is IRSAD, which is an indicator of Australian SES and was analyzed as part of the current article.

IRSAD summarizes information about the socioeconomic conditions of people and households within an area, including relative advantage and disadvantage measures. It is generally measured in decile on a score of 1–10. A low score indicates relatively greater disadvantage and a lack of advantage (*e.g.*, many households with low incomes or many people in unskilled occupations) and a high score indicates relatively greater advantage and a lack of disadvantage (*e.g.*, many households with high incomes or many people in skilled occupations).^21^ As per the Australian education system's report prepared by the Centre for International Research on Education Systems for the Mitchell Institute,^22^ young Australians generally represent three categories of SES: low (25%), medium (50%), high (25%). Taking this as a reference and based on the spread of our data, we categorized IRSAD score of deciles into three: 1 and 2 representing “low SES,” 3–7 as “medium SES,” and 8–10 as “high SES.”

### Study setting/location

#### Sites

The data linkage activity was performed independently by SA-NT Datalink, a nationwide consortium operating as part of the Population Health Research Network. De-identified data including SEIFA-IRSAD scores (SAPSC) and postcodes (CADOSA and NWAHS) were provided to the investigator team by SA-NT Datalink.

### Study population and setting

#### Data registries

##### Coronary Angiogram Database of South Australia

CADOSA is a statewide clinical quality registry of cardiac catheterization procedures performed in the public tertiary hospitals of South Australia (SA). The CADOSA registry^23^ collects data on a standardized case report form and is compatible with the American College of Cardiology CathPCI Registry^®^.^24^ This registry captures in-hospital data by prospective data collection on: sociodemographic, detailed clinical, angiographic, and outcome data on every SA public hospital patient undergoing coronary angiography/percutaneous coronary intervention. For SES comparative analyses, postcodes in the CADOSA registry were converted to SEIFA-IRSAD scores (deciles) using the 2016 ABS excel spread sheet accessible on their website.^25^ The data included for these comparative SES analyses was from January 01, 2012 to December 31, 2018.

##### South Australian Perinatal Statistics Collection

SAPSC is a publicly held state-owned database that records pregnancy and birth outcome data for all births in South Australia since 1981.^26^ Approximately there are 20,000 births per year in South Australia, and pregnancy data including SES indicators (such as SEIFA-IRSAD scores) are available in the SAPSC for >600,000 births since 1981, which signifies it being a highly robust registry. The data were made available from January 01, 1986 for this specific SES analysis. However, data for body mass index (BMI) were made available from 2007 onward and were limited to very few women.

##### North West Adelaide Health Study

NWAHS is a longitudinal biomedical population cohort study of ∼4000 adults, recruited from northern and western regions of Adelaide, South Australia during 1999–2003, with ongoing follow-up of participants.^27^ In addition, some of the lowest SES suburbs (such as Elizabeth South and Davoren Park) in South Australia, and even in Australia, are part of NWAHS.^21^ The study focuses on health conditions including asthma, diabetes, chronic obstructive pulmonary disease, arthritis, osteoporosis, mental health, sleep health, and CVD. NWAHS being a multidisciplinary dataset has been involved in several research projects both at international and national level. This is a privately held database. For SES comparative analyses, postcodes in the NWAHS registry were converted to SEIFA-IRSAD scores (deciles) using the 2016 ABS excel spread sheet accessible on their website.^25^

### Study objectives

The aim was to assess the role of SES on the association between pregnancy complications and PCAD through proxy indicators such as postcodes and SEIFA-IRSAD scores.

### Study procedures

#### Data linkage methodology

##### Ascertainment of cases

Cases were ascertained from CADOSA. Women <60 years in CADOSA having undergone coronary angiography, with obstructive CAD defined as ≥50% stenosis in one or more coronary arteries (defined as PCAD) were linked to SAPSC database to determine if they had a previous major pregnancy complication.

**Fig. 1.**
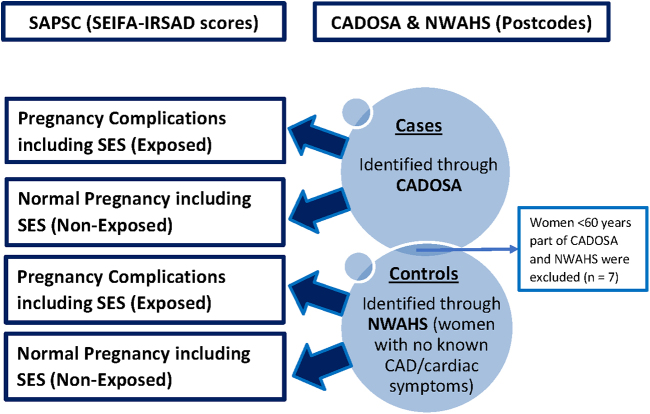
Case–control study design including SES comparative analyses—Diagrammatic illustration of the linkage process. SES, socioeconomic status.

Pregnancy complications included hypertensive disorders of pregnancy (HDP), which refers to a group of conditions mainly including gestational hypertension and preeclampsia (for this specific SAPSC cohort) that involves high blood pressure (≥140/90 mmHg) during pregnancy; preterm birth, which is defined as the spontaneous birth of a baby before 37 weeks of pregnancy; SGA infant, which is defined as having a birth weight <10th percentile for the gestational age; low birth weight (usually a combination of SGA and preterm birth), which is defined as the birth of a baby weighing <2,500 g; GDM, which is defined as glucose intolerance recognized first in pregnancy with revised diagnostic criteria of having fasting glucose of 5.6 mmol/g and/or 8.9 mmol/L in 60 minutes and/or 7.7 mmol/L in 120 minutes on 75 g of oral glucose tolerance test; or a history of previous miscarriage that is defined as loss of pregnancy before 20th week of gestation. Postcodes were also extracted from CADOSA (Fig. 1).

The SAPSC database was established in 1981 with its current dataset commencing in 1986. Therefore, the data were linked from January 01, 1986 to December 31, 2018. The first women recorded in SAPSC for this linkage are now approximately between 55 and 70 years of age. Key variables of interest in SAPSC included maternal age, preexisting medical conditions, socioeconomic indicators, pregnancy history, pregnancy complications, maternal smoking status, and BMI.

##### Ascertainment of controls

Controls were ascertained from the NWAHS registry. Women having health conditions or symptoms other than CVD/CAD (age-matched to CADOSA ±5 years) were linked to SAPSC database to identify their pregnancy outcomes (Fig. 1). Important variables extracted from NWAHS included general health and well-being, family history, diabetes, lung function, heart attack, stroke, angina, smoking, alcohol, and postcodes.

Women <60 years who were part of both CADOSA and NWAHS (*n* = 7) were excluded from the final analyses to prevent inclusion of any cases in the control group (Fig. 1).

### Data linkage management

The security practices and protocols at SA-NT Datalink have been developed in accordance with the Australian Government Protective Security Policy Framework. SA-NT Datalink is ISO 27001 certified. The privacy of the individual is protected by SA-NT Datalink's compliance with the Commonwealth Privacy Act (1988) and the South Australian Government Administrative Instruction: Information Privacy, Principles, Premier and Cabinet Circular 12 (2009). De-identified linked data were provided by SA-NT Datalink in the form of “cases” and “controls.”

### Statistical methods

The association between pregnancy complications and SEIFA-IRSAD scores from SAPSC was investigated using cross-tabulations with associated chi-square *p*-values or Fisher's exact test *p*-values as appropriate. The association between PCAD and SEIFA-IRSAD scores from SAPSC, CADOSA, and NWAHS was investigated using cross-tabulations with associated chi-square *p*-values. The association between PCAD and change in SEIFA-IRSAD scores was investigated using a cross-tabulation with associated chi-square *p*-value. Change in SEIFA-IRSAD scores was calculated using the difference in SEIFA-IRSAD scores at the time of either having PCAD (CADOSA) or no PCAD (NWAHS) and SEIFA-IRSAD scores during pregnancy (SAPSC).

Unadjusted binary logistic regression analyses were initially performed with outcome PCAD and predictor: SEIFA-IRSAD scores from SAPSC. Various covariates were then added to the model, one covariate at a time. Including only those covariates with *p* ≤ 0.25 on bivariate regression, an initial multivariable binary logistic model was created. Backward elimination was then performed until all covariates had *p* ≤ 0.05. This was the final parsimonious multivariable model for SEIFA-IRSAD scores from SAPSC as predictor.

Unadjusted binary logistic regression analyses were initially performed with outcome PCAD and predictor: change in SEIFA-IRSAD scores from CADOSA/NWAHS to SAPSC. Various covariates were then added to the model, one covariate at a time. Including only those covariates with *p* ≤ 0.25 on bivariate regression, an initial multivariable binary logistic model was created. Backward elimination was then performed until all covariates had *p* ≤ 0.05. This was the final parsimonious multivariable model for change in SEIFA-IRSAD scores as predictor.

### Ethics approvals

Ethics approvals from the following Human Research Ethics Committees (HRECs) were obtained:

- South Australian Department for Health and Wellbeing HREC, HREC/20/SAH/51
- HERC of the University of Adelaide, Approval ID: 34704

## Results

The number of records for SEIFA-IRSAD scores for women in SAPSC was *n* = 914, whereas number of records for SEIFA-IRSAD scores for women in CADOSA and NWAHS was *n* = 913.

### Association between pregnancy complications and SES during pregnancy

Among the placenta-mediated pregnancy complications, preterm birth was independently and significantly associated with low SES as reflected by SEIFA-IRSAD scores at the time of pregnancy (*p* < 0.001). None of the other pregnancy complications were associated with SES, although low birth weight was marginally insignificantly associated with low SES (*p* = 0.067). Of the CVD risk factors, any cigarette smoking during pregnancy was significantly associated with low SES (*p* < 0.001). Of note, women who had preexisting medical conditions during pregnancy were not associated with SES (Table 1). In a nutshell, women who had preterm delivery or were smokers were independently associated with having low SES during pregnancy.

**Table 1.** Independent Association Between Pregnancy Complications and Socioeconomic Status During Pregnancy

Risk factors including pregnancy complications	SEIFA-IRSAD scores^a^	*Risk factors^b^ present (yes) * *n* * (%)*	*Risk factors absent (no) *n* (%)*	*p^c^*
Placenta-mediated pregnancy complications				
Hypertensive disorder of pregnancy	SES high	6 (15.4)	109 (12.5%)	0.86
	SES medium	16 (41.0)	378 (43.2%)	
	SES low	17 (43.6)	388 (44.3%)	
Preterm birth (<37 weeks)	SES high	25 (12.0)	90 (12.8)	<0.001^c^
	SES medium	64 (30.8)	330 (46.8)	
	SES low	119 (57.2)	285 (40.4)	
Low birth weight (<2,500 g)	SES high	27 (12.9)	88 (12.5)	0.067
	SES medium	76 (36.4)	318 (45.1)	
	SES low	106 (50.7)	299 (42.4)	
Gestational diabetes mellitus	SES high	1 (3.1)	114 (12.9)	0.266
	SES medium	15 (46.9)	379 (43.0)	
	SES low	16 (50.0)	389 (44.1)	
Small for gestational age	SES high	5 (17.2)	110 (12.4)	0.74
	SES medium	12 (41.4)	382 (43.2)	
	SES low	12 (41.4)	393 (44.4)	
Other pregnancy complications				
History of a previous miscarriage	SES high	13 (12.3)	28 (14.4)	0.551
	SES medium	46 (43.4)	93 (47.7)	
	SES low	47 (44.3)	74 (37.9)	
Previous still birth	SES high	0 (0)	41 (14.0)	0.087
	SES medium	2 (22.2)	137 (47.0)	
	SES low	7 (77.8)	114 (39.0)	
Medical conditions during pregnancy				
Diabetes type 1 and 2 (preexisting)	SES high	5 (21.8)	110 (12.4)	0.284
	SES medium	7 (30.4)	387 (43.4)	
	SES low	11 (47.8)	394 (44.2)	
Hypertension (preexisting)	SES high	8 (17.0)	107 (12.3)	0.497
	SES medium	17 (36.2)	377 (43.5)	
	SES low	22 (46.8)	383 (44.2)	
Asthma	SES high	5 (12.2)	110 (12.6)	0.742
	SES medium	20 (48.8)	374 (42.8)	
	SES low	16 (39.0)	389 (44.6)	
Anemia	SES high	16 (14.1)	99 (12.3)	0.294
	SES medium	41 (36.3)	353 (44.1)	
	SES low	56 (49.6)	349 (43.6)	
Other CVD risk factors during pregnancy				
Any amount of cigarette smoking per day	SES high	9 (7.7)	31 (17.7)	<0.001^c^
	SES medium	47 (40.2)	91 (52.0)	
	SES low	61 (52.1)	53 (30.3)	
Overweight + Obese (BMI ≥25 kg/m^2^)	SES high	4 (8.0)	3 (15.8)	0.355
	SES medium	27 (54.0)	7 (36.8)	
	SES low	19 (38.0)	9 (47.4)	

^a^ SES divided into: high, medium, and low reflective of SEIFA-IRSAD scores.

^b^ Presence of risk factors for premature coronary artery disease including pregnancy complications, medical conditions during pregnancy, and other cardiovascular risk factors during pregnancy.

^c^ *p* ≤ 0.05 was taken as being statistically significant.

BMI, body mass index; CVD, cardiovascular disease; SEIFA-IRSAD, socioeconomic index for areas–index of relative socio-economic advantage and disadvantage; SES, socioeconomic status.

### Association between PCAD and SES

When stratified by SES during pregnancy, the prevalence of PCAD was 11.9% in women with high SES compared with 47.1% among those with low SES and this association was statistically significant (*p* = 0.005). When stratified by SES at the time of diagnosis of PCAD, the prevalence of PCAD was 16.1% among women with high SES compared with 40.9% among those with low SES but, this association was not significant (Table 2). This meant that women who had low SES at the time of pregnancy were significantly associated with PCAD risk later in life.

**Table 2.** Association Between Premature Coronary Artery Disease and Socioeconomic Status During Pregnancy and at the Time of Premature Coronary Artery Disease Diagnosis

SEIFA-IRSAD scores during pregnancy^a^	*PCAD yes, n* (%)	*PCAD no, n* (%)	*p^b^*
SES high	86 (11.9)	29 (15.0)	0.005
SES medium	295 (41.0)	99 (51.0)	
SES low	339 (47.1)	66 (34.0)	
SEIFA-IRSAD scores at the time of PCAD diagnosis^a^			
SES high	116 (16.1)	22 (11.3)	0.236
SES medium	309 (43.0)	91 (46.9)	
SES low	294 (40.9)	81 (41.8)	

^a^ SES divided into high, medium, and low reflective of SEIFA-IRSAD scores from SAPSC (during pregnancy), CADOSA and NWAHS (at the time of PCAD diagnosis) respectively.

^b^ *p* ≤ 0.05 was taken as being statistically significant.

CADOSA, Coronary Angiogram Database of South Australia; NWAHS, North West Adelaide Health Study; PCAD, premature coronary artery disease; SAPSC, South Australian Perinatal Statistics Collection.

#### Association between PCAD and Change in SES from pregnancy to time of diagnosis of PCAD

Prevalence of PCAD in women with improvement in SES from pregnancy to time of diagnosis of PCAD was 47.6% compared with prevalence of PCAD with decrement in SES was 36.2% and this association was statistically significant (*p* = 0.040; Table 3). However, pregnancy complications such as preterm birth (61.3% vs. 38.7%), GDM (88.5% vs. 11.5%), and cardiovascular risk factor, that is, smoking (65.4% vs. 34.6%), were significantly more prevalent in women with improved SES compared with women with decreased SES (*p* = 0.039, <0.001, and 0.001, respectively; Table 4). Table 3 concluded that women who had improvement in SES from pregnancy till PCAD diagnosis had higher prevalence of PCAD and Table 4 highlighted that preterm birth, GDM, and smoking were more prevalent in women with improved SES.

**Table 3.** Association Between Premature Coronary Artery Disease Versus Change in Socioeconomic Status Scores

Change in SEIFA-IRSAD scores^a^	*PCAD yes, *n* (%)*	*PCAD no, *n* (%)*	*p^b^*
SES improved	342 (47.6)	75 (38.7)	0.040
SES same	116 (16.2)	30 (15.4)	
SES decreased	260 (36.2)	89 (45.9)	

^a^ SES divided into high, medium, and low reflective of change in SEIFA-IRSAD scores from SES at the time of having PCAD (CADOSA) or no PCAD (NWAHS) minus the SES at the time of pregnancy (SAPSC).

^b^ *p* ≤ 0.05 was taken as being statistically significant.

**Table 4.** Association Between Pregnancy Complications and Change in Socioeconomic Status from Pregnancy to Time of Diagnosis of Premature Coronary Artery Disease

Risk factors including pregnancy complications	Change in SES scores^a^	*Risk factor^b^ present (yes), * *n* * (%)*	*Risk factor, absent (no), * *n* * (%)*	*p* ^c^
Placenta mediated pregnancy complications				
Hypertensive disorder of pregnancy	SES improved	22 (66.7)	395 (53.9)	0.149
	SES decreased	11 (33.3)	338 (46.1)	
Preterm birth (<37 weeks)	SES improved	106 (61.3)	310 (52.4)	0.039^c^
	SES decreased	67 (38.7)	282 (47.6)	
Low birth weight (<2,500 g)	SES improved	103 (57.9)	314 (53.4)	0.295
	SES decreased	75 (42.1)	274 (46.6)	
Gestational diabetes mellitus	SES improved	23 (88.5)	394 (53.2)	<0.001^c^
	SES decreased	3 (11.5)	346 (46.8)	
Small for gestational age	SES improved	11 (50.0)	406 (54.6)	0.67
	SES decreased	11 (50.0)	338 (45.4)	
Medical conditions during pregnancy				
Diabetes type 1 and 2 (preexisting)	SES improved	11 (55.0)	406 (54.4)	0.96
	SES decreased	9 (45.0)	340 (45.6)	
Hypertension (preexisting)	SES improved	21 (48.8)	396 (54.8)	0.448
	SES decreased	22 (51.2)	327 (45.2)	
Other CVD risk factors during pregnancy				
Any cigarette smoking	SES improved	70 (65.4)	69 (45.4)	0.001^c^
	SES decreased	37 (34.6)	83 (54.6)	
Overweight + obese (BMI ≥25 kg/m^2^)	SES improved	9 (56.3)	26 (57.8)	0.915
	SES decreased	7 (43.7)	19 (42.2)	

^a^ SES divided into high, medium, and low reflective of change in SEIFA-IRSAD scores from SES at the time of having PCAD (CADOSA) or no PCAD (NWAHS) minus the SES at the time of pregnancy (SAPSC).

^b^ Presence of risk factors for premature coronary artery disease including pregnancy complications, medical conditions during pregnancy, and other cardiovascular risk factors during pregnancy.

^c^ p ≤ 0.05 was taken as being statistically significant.

### Final modeling of PCAD versus SES and change in SES scores

#### PCAD versus SES during pregnancy and significant risk factors

Table 5 shows that low SES during pregnancy was associated with the development of early CAD (unadjusted *p* = 0.005). However, the final model revealed that the association between low SES during pregnancy and PCAD was not significant after adjusting for confounders such as age, history of miscarriage, preterm birth (*i.e.*, <37 weeks), and any cigarette smoking during pregnancy. For Table 5, SES at the time of pregnancy was independently associated with development of PCAD; however, this association was insignificant when adjusted for confounders mentioned previously.

**Table 5.** Association of Premature Coronary Artery Disease and Socioeconomic Status During Pregnancy Including Significant Risk Factors

Adjustment	Predictor	Comparison	Odds ratio^a^ with 95% confidence interval	Comparison, *p*-value	Global *p*-value^b^
Unadjusted	SEIFA-IRSAD scores (at the time of pregnancy)	• High SES versus medium SES	1.00 (0.62–1.61)	0.984	0.005
		• High SES versus low SES	0.58 (0.35–0.95)	0.030	
		• Medium SES versus low SES	0.58 (0.41–0.82)	0.002	
Adjusted	SEIFA-IRSAD scores (at the time of pregnancy)	• High SES versus medium SES	1.07 (0.45–2.52)	0.879	0.285
		• High SES versus low SES	0.59 (0.22–1.57)	0.290	
		• Medium SES versus low SES	0.55 (0.26–1.16)	0.118	
	Age during pregnancy	Per 1 year increase	0.93 (0.87–1.00)		0.037
	Previous miscarriage	Yes versus No	2.14 (1.02–4.49)		0.044
	Preterm birth	<37 weeks versus ≥37 weeks	4.77 (1.74–13.03)		0.002
	Average cigarettes per day during pregnancy	Any smoking versus No smoking	8.60 (3.25–22.75)		<0.001

^a^ Modeling the probability of having premature coronary artery disease using multivariable binary logistic regression analysis.

^b^ *p* ≤ 0.05 was taken as being statistically significant.

#### PCAD versus change in SES scores and significant risk factors

Table 6 demonstrates that an improvement in SES score was associated with the increased risk of development of early CAD (unadjusted *p* = 0.041). However, the final model revealed that the association between improvement in SES and PCAD was not significant after adjusting for confounders such as age, history of miscarriage, preterm birth, that is, <37 weeks, and any cigarette smoking during pregnancy. For Table 6, improvement in SES (*i.e.*, from pregnancy to PCAD diagnosis) was associated with increased risk of PCAD; however, when adjusted with other risk factors, the association between change in SES scores and development of PCAD was insignificant.

**Table 6.** Association of Premature Coronary Artery Disease and Change in Socioeconomic Status Scores Including Significant Risk Factors

Adjustment	Predictor	Comparison	Odds ratio^a^ with 95% confidence interval	*Comparison * *p* *-value*	*Global * *p* *-value^b^*
Unadjusted	Change in SEIFA-IRSAD scores between SES at the time of having PCAD/no PCAD and SES at the time of pregnancy	• SES decreased versus SES same	0.76 (0.47–1.21)	0.240	0.041
		• SES same versus SES improved	1.18 (0.73–1.89)	0.494	
		• SES improved versus SES decreased	1.56 (1.10–2.21)	0.012	
Adjusted	Change in SEIFA-IRSAD scores between SES at the time of having PCAD/no PCAD and SES at the time of pregnancy	• SES decreased versus SES same	0.56 (0.19–1.69)	0.305	0.425
		• SES same versus SES improved	0.80 (0.26–2.48)	0.705	
		• SES improved versus SES decreased	1.43 (0.74–2.80)	0.290	
	Age during pregnancy	Per 1 year increase	0.92 (0.87–0.99)		0.020
	Previous miscarriage	Yes versus No	2.09 (1.00–4.35)		0.050
	Preterm birth	<37 weeks versus ≥37 weeks	4.40 (1.61–12.05)		0.004
	Average cigarettes per day during pregnancy	Any smoking versus no smoking	8.75 (3.32–23.07)		<0.001

^a^ Modeling the probability of having premature coronary artery disease using multivariable binary logistic regression analysis.

^b^ *p* ≤ 0.05 was taken as being statistically significant.

## Discussion

To the best of our knowledge, this is the first study to report the role of SES on the association between pregnancy complications and PCAD. This data linkage study showed that SES at the time of pregnancy and change in SES when adjusted for pregnancy complications and smoking were not significantly associated with PCAD. This translates that a woman with low SES who is a nonsmoker (living a healthy lifestyle) and also had a totally uncomplicated pregnancy does not have an increased risk of PCAD which seems clinically quite relevant.

Four markers of SES were shown to be associated with CVD risk in high-income countries including income level, educational attainment, employment status, and environmental factors, the last one representing SES characteristics at the population level.^28–30^ A longitudinal cohort study conducted in Southern Alberta, Canada, showed that residence in a neighborhood with low SES plays an important role in accessing cardiac health services leading to adverse cardiac outcomes, especially in women compared with men.^31^ Similar to the findings from the literature, our data linkage study demonstrated that SES score at the time of pregnancy was significantly associated with PCAD risk.

Furthermore, when change in SES scores was considered, a woman with an improved SES score (*i.e.*, moving from a low SES postcode area to a high SES postcode area) still had high prevalence of PCAD later in life. This appears to be associated with a high prevalence of the unmodifiable risk factors of pregnancy complications including preterm birth and GDM and her persistent unhealthy lifestyle such as smoking when moving to a high SES suburb.

Women who give birth preterm are at twice the risk of CAD compared with those who give birth at term.^5^ The four main pathways leading to preterm birth include ascending genital infections (disturbed microbiome), placental dysfunction, stress, and excessive uterine stretch.^32,33^ In particular placental dysfunction and ascending infections/disturbed microbiome can be associated with inflammatory cytokines and dyslipidemia, both of which are associated with atherosclerosis and endothelial dysfunction leading to increased CVD risk.^34^ A study from Korea utilizing their national health insurance database revealed that mothers in the low SES group had higher rates of preterm delivery compared with those in the middle or high SES groups.^16^ Extended working hours and work-related fatigue have been identified as risk factors for preterm delivery.^35^

Our findings are consistent with those of the Korean study and demonstrate that in both the final models women who deliver preterm are at four-fold increased risk of PCAD after adjusting for SES scores at the time of pregnancy. For these cohorts, preterm birth underlines the risk for PCAD and moving to a higher SES area does not mitigate the risk. This may be because of the fact that these women had higher prevalence of pregnancy complications such as HDP, low birth weight, GDM, and had preexisting diabetes (type 1 or 2), were smokers and obese, despite moving to a higher SES area.

Miscarriage is one of the most common adverse pregnancy outcome defined as the loss of pregnancy before 20 weeks of gestation,^36^ majority being in the first trimester of pregnancy.^37^ Miscarriage is a heterogeneous entity and it is difficult to identify a single pathological cause; however, the most common causes include chromosomal abnormalities such as aneuploidy, methylenetetrahydrofolate reductase polymorphism that is associated with high homocysteine levels, maternal age, abnormal embryo development, infections such as cytomegalovirus or rubella, idiopathic or poorly controlled diabetes, and auto-immune disorders such as antiphospholipid syndrome and systemic lupus erythematosus.^38–42^ Regarding the association between history of miscarriage and CVD, some shared risk factors such as smoking, excessive alcohol intake, and obesity play important roles as confounders in the causal pathway. After adjusting for these risk factors, a stronger evidence for an association between miscarriage and future CVD risk in mothers was found.^43^

In a survey conducted in Lahore, Pakistan, the study concluded that having low SES was indirectly associated with miscarriage, because improper diet was the main reason for pregnancy loss before 20 weeks, the latter being associated with poverty among pregnant women.^44^ Similarly, a Korean Prenatal Diagnosis Study conducted in Seoul from 2016 to 2018 revealed that there was an inverse association between the risk of fetal chromosomal abnormality leading to miscarriage and low level of household income in a prospective cohort of pregnant women.^45^ Our current data linkage study (for both the final models) also showed that women with a history of miscarriage are twice as likely to experience PCAD compared with women without any pregnancy loss after adjusting for SES scores at the time of pregnancy.

GDM is strongly associated with the lifetime development of CAD even in the absence of overt type 2 diabetes mellitus.^46,47^ A systematic review of observational studies found that women with a history of GDM have a 45% increased risk of CAD compared with women without GDM.^48^ The relationship between SES and GDM has been studied in various contexts and it has been shown that low SES is associated with an increased risk of GDM in pregnant women and vice versa.^49–51^ This relationship tends to become stronger in the presence of advanced maternal age and increase prepregnancy BMI.^51^ The findings of our study revealed that GDM is associated with an increased risk of PCAD and even moving to a higher SES suburb does not reduce that risk. This can be owing to increased prevalence of pregnancy complications and preexisting diabetes (type 1 or 2) in these women as mentioned previously.

Smoking is considered as a major risk factor for influencing the pattern (location of the damaged artery) and severity of CAD.^52^ Cigarette smoking causes a wide range of vascular abnormalities including dyslipidemia, endothelial dysfunction, defects in coagulation and fibrinolysis, and platelet dysfunction.^53^ Maternal smoking during pregnancy has proven to be associated with an increased risk of maternal CVD and the risk was higher for mothers who smoked during their last pregnancy.^54^ In addition, smoking during pregnancy has not only been linked to adverse pregnancy outcomes such as low birth weight, SGA, and preterm birth but also has an inverse association between SES and the frequency of maternal smoking during pregnancy.^55,56^ Income level has been significantly associated with CVD risk and a large study representing United States and Finland found a similar association with an increased risk of myocardial infarction and cardiac mortality in low-income cohorts after adjusting for smoking.^57^

Findings of our study are similar to the preexisting literature: mothers who smoke during pregnancy are eight times more likely to experience PCAD (as shown in both the final models) compared with mothers who are nonsmokers after adjusting for SES scores at the time of pregnancy. One of the reasons could be that many women who smoke at conception give up at least while they are pregnant but those who continue to smoke during pregnancy are more likely to continue smoking in to later life, even if these women move to a higher SES area, indicating it is a strong behavioral risk factor.

### Strengths and limitations of this study

The use of three robust, comprehensive databases as part of this linkage project provided a unique opportunity to assess the role of SES on the association between pregnancy complications and PCAD in Australian women. This data linkage activity is the first of its kind in Australia using a control cohort and was performed independently by a third party, SA-NT Datalink. Researchers were involved in data analyses and interpretation and had access to de-identified data only, thereby minimizing selection bias. One-to-one comparison of women representing SEIFA-IRSAD scores (in deciles) across all three databases, served as ideal “Cases” and “Controls.”

One of the limitations of this study was owing to it being a retrospective case–control study; causality could not be established between pregnancy complications (including other CVD risk factors) and PCAD after adjusting for SES, although temporal inference could be drawn. Second, because we were interested in one-to-one comparison of women across three databases, women with multiple pregnancies, having maximum number of pregnancy complications in a single pregnancy were included, while omitting other pregnancies, reducing our overall sample size for this data linkage activity. In addition, data for BMI were collected from 2007 onward in SAPSC database, which restricted our numerator (to *n* = 69) of the total women included in this cohort. Third, we had the issue of missing data in SAPSC database because some of the risk factors for both pregnancy complications and CVD were included in the database across time rather than from the onset of data collection.

Fourth, SEIFA-IRSAD scores are used as proxy indicator for SES within Australian communities and they represent population level SES characteristics (ranking areas into relative socioeconomic advantage and disadvantage) instead of individual level data. These proxy indicators might tend to misclassify SES at the individual level. Fifth, all the three databases (CADOSA, NWAHS, and SAPSC) might have had the issue of reporting bias (or recall bias) because self-reported data such as “average number of cigarettes smoked per day” was collected from respective study participants. Sixth, SAPSC database did not differentiate spontaneous from iatrogenic preterm birth and also gestational hypertension from preeclampsia. As a result, our findings are limited to women with preterm birth and HDP in general.

## Conclusion

This data linkage study found that women's SES at the time of pregnancy was not associated with PCAD. In addition, women diagnosed with PCAD have conventional CVD risk factor such as smoking and some that are uniquely related to their pregnancy (maternal placental syndromes)^58^ such as preterm birth and miscarriage in the presence of maternal age and SES. This calls for improved recognition by the medical and midwifery community of these clinical risk factors for future PCAD as part of preventative cardiology practice. These women may benefit from postpartum follow-up to increase their awareness of the risk of PCAD, encourage healthy lifestyle, increase access to health care for women, and manage CVD risk factors as they appear in the future to reduce PCAD risk.^59^ Moreover, these findings also provide evidence for the need for targeted postpartum interventions for women who experience pregnancy complication such as preterm delivery.

## Data Availability

On reasonable request, the data used to support this study's findings can be obtained from the corresponding author, A.K. via e-mail: adeel.khoja@adelaide.edu.au

## Abbreviations Used

ABS: Australian Bureau of Statistics
BMI: body mass index
CAD: coronary artery disease
CADOSA: Coronary Angiogram Database of South Australia
CVD: cardiovascular disease
CI: confidence interval
GDM: gestational diabetes mellitus
HDP: hypertensive disorders of pregnancy
HRECs: Human Research Ethics Committees
NWAHS: North West Adelaide Health Study
OR: odds ratio
PCAD: premature coronary artery disease
SA: South Australia
SAPSC: South Australian Perinatal Statistics Collection
SEIFA-IRSAD: socioeconomic indexes for areas–index of relative socioeconomic advantage and disadvantage
SES: socioeconomic status
SGA: small for gestational age

## References

[1] Scanlon PJ, Faxon DP, Audet A-M, et al. ACC/AHA guidelines for coronary angiography: executive summary and recommendations: A report of the American College of Cardiology/American Heart Association Task Force on Practice Guidelines (Committee on Coronary Angiography) developed in collaboration with the Society for Cardiac Angiography and Interventions. Circulation 1999;99:2345–2357; doi: 10.1161/01.CIR.99.17.2345 10226103

[2] Abdul-Razak S, Rahmat R, Mohd Kasim A, et al. Diagnostic performance of various familial hypercholesterolaemia diagnostic criteria compared to Dutch lipid clinic criteria in an Asian population. BMC Cardiovasc Disord 2017;17:1–8; doi: 10.1186/s12872-017-0694-z 29037163 PMC5644062

[3] Cook NR, Paynter NP, Eaton CB, et al. Comparison of the Framingham and Reynolds Risk scores for global cardiovascular risk prediction in the multiethnic Women's Health Initiative. Circulation 2012;125:1748–1756; doi: 10.1161/CIRCULATIONAHA.111.075929 22399535 PMC3324658

[4] Hermes W, Tamsma JT, Grootendorst DC, et al. Cardiovascular risk estimation in women with a history of hypertensive pregnancy disorders at term: A longitudinal follow-up study. BMC Pregnan Childbirth 2013;13:1–11; doi: 10.1186/1471-2393-13-126 PMC3680191 23734952

[5] Andraweera P, Dekker G, Arstall M, et al. Complications of Pregnancy and Future Cardiovascular Risk. In: Encyclopedia of Cardiovascular Research and Medicine, Vol. 1. (Vasan RS, Sawyer DB., eds.). Elsevier, Inc.: Oxford, 2018; pp. 643–650; doi.org/10.1016/B978-0-12-809657-4.99726-6

[6] Archambault C, Arel R, Filion KB. Gestational diabetes and risk of cardiovascular disease: A scoping review. Open Med 2014;8:e1–e9. 25009679 PMC4085089

[7] Bellamy L, Casas J-P, Hingorani AD, et al. Type 2 diabetes mellitus after gestational diabetes: A systematic review and meta-analysis. Lancet 2009;373:1773–1779; doi: 10.1016/S0140-6736(09)60731-5 19465232

[8] Fraser A, Nelson SM, Macdonald-Wallis C, et al. Associations of pregnancy complications with calculated CVD risk and cardiovascular risk factors in middle age: The Avon Longitudinal Study of Parents and Children. Circulation 2012;125:1367; doi: 10.1161/CIRCULATIONAHA.111.044784 22344039 PMC3323835

[9] Mosca L, Benjamin EJ, Berra K, et al. Effectiveness-based guidelines for the prevention of cardiovascular disease in women—2011 update: A guideline from the American Heart Association. Circulation 2011;123:1243–1262; doi: 10.1161/CIR.0b013e31820faaf8 21325087 PMC3182143

[10] Fuster V, Kelly BB, Vedanthan R. Global cardiovascular health: Urgent need for an intersectoral approach. J Am Coll Cardiol 2011;58:1208–1210; doi: 10.1016/j.jacc.2011.05.038 21903051

[11] Heida KY, Bots ML, De Groot CJ, et al. Cardiovascular risk management after reproductive and pregnancy-related disorders: A Dutch multidisciplinary evidence-based guideline. Eur J Prev Cardiol 2016;23:1863–1879; doi: 10.1177/2047487316659573 27432836

[12] Liu L, Oza S, Hogan D, et al. Global, regional, and national causes of child mortality in 2000–2013, with projections to inform post-2015 priorities: An updated systematic analysis. Lancet 2015;385:430–440; doi: 10.1016/S0140-6736(14)61698-6 25280870

[13] Oliver-Williams CT, Heydon EE, Smith GC, et al. Miscarriage and future maternal cardiovascular disease: A systematic review and meta-analysis. Heart 2013;99:1636–1644; doi: 10.1136/heartjnl-2012-303237 23539554 PMC3812894

[14] Australian Institute of Health and Welfare. Australia's Mothers and Babies 2015—In Brief. Perinatal Statistics Series No. 33. Cat. No. PER 91. AIHW: Canberra; 2017. Available from: https://www.aihw.gov.au/reports/mothersbabies/australias-mothers-babies-2015-in-brief/summary [Last accessed: December 01, 2023].

[15] Joseph K, Liston RM, Dodds L, et al. Socioeconomic status and perinatal outcomes in a setting with universal access to essential health care services. CMAJ 2007;177:583–590; doi: 10.1503/cmaj.061198 17846440 PMC1963370

[16] Kim MK, Lee SM, Bae S-H, et al. Socioeconomic status can affect pregnancy outcomes and complications, even with a universal healthcare system. Int J Equity Health 2018;17:1–8; doi: 10.1186/s12939-017-0715-7 29304810 PMC5756361

[17] Shaw LJ, Bairey Merz CN, Bittner V, et al. Importance of socioeconomic status as a predictor of cardiovascular outcome and costs of care in women with suspected myocardial ischemia. Results from the National Institutes of Health, National Heart, Lung and Blood Institute-sponsored Women's Ischemia Syndrome Evaluation (WISE). J Womens Health 2008;17:1081–1092; doi: 10.1089/jwh.2007.0596 PMC2818766 18774893

[18] William MS, Heval MK, John CL, et al. Socioeconomic status and cardiovascular outcomes. Circulation 2018;137:2166–2178; doi: 10.1161/CIRCULATIONAHA.117.029652 29760227 PMC5958918

[19] Stringhini S, Carmeli C, Jokela M, et al. Socioeconomic status and the 25 × 25 risk factors as determinants of premature mortality: A multicohort study and meta-analysis of 17 million men and women. Lancet 2017;389:1229–1237; doi: 10.1016/S0140-6736(16)32380-7 28159391 PMC5368415

[20] Minhas AMK, Jain V, Li M, et al. Family income and cardiovascular disease risk in American adults. Sci Rep 2023;13:279; doi: 10.1038/s41598-023-27474-x 36609674 PMC9822929

[21] Socio-Economic Indexes for Areas (SEIFA). 2016. Available from: https://www.abs.gov.au/websitedbs/censushome.nsf/home/seifa [Last accessed: March 10, 2023].

[22] Lamb S, Huo S, Walstab A, et al. Educational Opportunity in Australia 2020: Who Succeeds and Who Misses Out. Available from: https://vuir.vu.edu.au/42362/1/educational-opportunity-in-australia-2020.pdf [Last accessed: March 10, 2023].

[23] Arora J, Tavella R. Implementing ICHOM's standard sets of outcomes: Coronary artery disease in the coronary angiogram database of South Australia (CADOSA). London: UK Int Consort Heal Outcomes Meas 2017:1–16.

[24] Baumann AA, Tavella R, Air TM, et al. Prevalence and real-world management of NSTEMI with multivessel disease. Cardiovascular Diagnosis and Therapy 2022;12:1; doi: 10.21037/cdt-21-518 35282665 PMC8898694

[25] Census of Population and Housing: Socio-Economic Indexes for Areas (SEIFA), Australia; 2016. Available from: https://www.abs.gov.au/AUSSTATS/abs@.nsf/DetailsPage/2033.0.55.0012016?OpenDocument [Last accessed: March 10, 2023].

[26] South Australian Mother and Babies. 2018. Available from: https://data.sa.gov.au/data/dataset/south-australian-perinatal-statistics-collection [Last accessed: March 10, 2023].

[27] Grant JF, Chittleborough CR, Taylor AW, et al. The North West Adelaide Health Study: Detailed methods and baseline segmentation of a cohort for selected chronic diseases. Epidemiol Persp Innov 2006;3:1–14; doi: 10.1186/1742-5573-3-4 PMC1462963 16608529

[28] Clark CR, Ommerborn MJ, Hickson DA, et al. Neighborhood disadvantage, neighborhood safety and cardiometabolic risk factors in African Americans: Biosocial associations in the Jackson Heart study. PLoS One 2013;8:e63254; doi: 10.1371/journal.pone.0063254 23691005 PMC3653956

[29] Diez Roux AV. Residential environments and cardiovascular risk. J Urban Health 2003;80:569–589; doi: 10.1093/jurban/jtg065 14709706 PMC3456219

[30] Roux AVD, Merkin SS, Arnett D, et al. Neighborhood of residence and incidence of coronary heart disease. N Engl J Med 2001;345:99–106; doi: 10.1056/NEJM200107123450205 11450679

[31] Fabreau GE, Leung AA, Southern DA, et al. Sex, socioeconomic status, access to cardiac catheterization, and outcomes for acute coronary syndromes in the context of universal healthcare coverage. Circ Cardiovasc Qual Outcomes 2014;7:540–549; doi: 10.1161/CIRCOUTCOMES.114.001021 24895450 PMC4411180

[32] Bayar E, Bennett PR, Chan D, et al. The Pregnancy microbiome and preterm birth. Semin Immunopathol 2020;42:487–499; doi: 10.1007/s00281-020-00817-w 32797272 PMC7508933

[33] Staude B, Oehmke F, Lauer T, et al. The microbiome and preterm birth: A change in paradigm with profound implications for pathophysiologic concepts and novel therapeutic strategies. BioMed Res Int 2018;2018:7218187; doi: 10.1155/2018/7218187 30370305 PMC6189679

[34] Catov JM, Bodnar LM, Ness RB, et al. Inflammation and dyslipidemia related to risk of spontaneous preterm birth. Am J Epidemiol 2007;166:1312–1319; doi: 10.1093/aje/kwm273 17906337

[35] Luke B, Mamelle N, Keth L, et al. The association between occupational factors and preterm birth: A United States nurses' study. Am J Obstet Gynecol 1995;173:849–862; doi: 10.1016/0002-9378(95)90354-2 7573257

[36] Bhattacharya S, Townend J, Shetty A, et al. Does miscarriage in an initial pregnancy lead to adverse obstetric and perinatal outcomes in the next continuing pregnancy? BJOG 2008;115:1623–1629; doi: 10.1111/j.1471-0528.2008.01943.x 18947339

[37] Wang X, Chen C, Wang L, et al. Conception, early pregnancy loss, and time to clinical pregnancy: A population-based prospective study. Fertil Steril 2003;79:577–584; doi: 10.1016/s0015-0282(02)04694-0 12620443

[38] Garcıa-Enguıdanos A, Calle ME, Valero J, et al. Risk factors in miscarriage: A review. Eur J Obstet Gynecol Reprod Biol 2002;102:111–119; doi: 10.1016/s0301-2115(01)00613-3 11950476

[39] Smith G, Wood A, Pell J, et al. Recurrent miscarriage is associated with a family history of ischaemic heart disease: A retrospective cohort study. BJOG 2011;118:557–563; doi: 10.1111/j.1471-0528.2010.02890.x 21244619

[40] Larsen EC, Christiansen OB, Kolte AM, et al. New insights into mechanisms behind miscarriage. BMC Med 2013; 11: 1–10; doi: 10.1186/1741-7015-11-154 23803387 PMC3699442

[41] Jia C-W, Wang L, Lan Y-L, et al. Aneuploidy in early miscarriage and its related factors. Chin Med J 2015;128:2772–2776; doi: 10.4103/0366-6999.167352 26481744 PMC4736891

[42] Merviel P, Cabry R, Lourdel E, et al. Comparison of two preventive treatments for patients with recurrent miscarriages carrying a C677T methylenetetrahydrofolate reductase mutation: 5-year experience. J Int Med Res 2017;45:1720–1730; doi: 10.1177/0300060516675111 28703660 PMC5805189

[43] Smith GC, Pell JP, Walsh D. Spontaneous loss of early pregnancy and risk of ischaemic heart disease in later life: Retrospective cohort study. BMJ 2003;326:423–424; doi: 10.1136/bmj.326.7386.423 12595381 PMC149442

[44] Malik SIA, Shahzad L, Hussain S, et al. How poverty, malnutrition and other socioeconomic factors affect maternal health: A quantitative study from Lahore Pakistan. Pakistan J Med Health Sci 2022;16:230–230; doi: 10.53350/pjmhs22169230

[45] Choe S-A, Lee SM, Han YJ, et al. Chromosomal abnormality, fetal/neonatal death and socioeconomic status: A Prospective Cohort Study. Matern Child Health J 2023;27:111–116; doi: 10.1007/s10995-022-03542-y 36352289

[46] Pathirana MM, Lassi Z, Ali A, et al. Cardiovascular risk factors in women with previous gestational diabetes mellitus: A systematic review and meta-analysis. Rev Endoc Metab Disord 2021;22:729–761; doi: 10.1007/s11154-020-09587-0 33106997

[47] Kramer CK, Campbell S, Retnakaran R. Gestational diabetes and the risk of cardiovascular disease in women: A systematic review and meta-analysis. Diabetologia 2019;62:905–914; doi: 10.1007/s00125-019-4840-2 30843102

[48] Xie W, Wang Y, Xiao S, et al. Association of gestational diabetes mellitus with overall and type specific cardiovascular and cerebrovascular diseases: Systematic review and meta-analysis. BMJ 2022;378; doi: 10.1136/bmj-2022-070244 PMC9490552 36130740

[49] Song L, Shen L, Li H, et al. Socio-economic status and risk of gestational diabetes mellitus among Chinese women. Diabetic Med 2017;34:1421–1427; doi: 10.1111/dme.13415 28636764

[50] Liu J, Liu E, Leng J, et al. Indicators of socio-economic status and risk of gestational diabetes mellitus in pregnant women in urban Tianjin, China. Diabetes Res Clin Pract 2018;144:192–199; doi: 10.1016/j.diabres.2018.08.023 30205183

[51] Al-Shaikh G, Alshaikh M, Al-Shaikh R, et al. Association of socio economic status with gestational diabetes mellitus among Saudi Women. La Prensa Med Argentina 2016;102:2; doi: 10.4172/lpma.1000195

[52] Salehi N, Janjani P, Tadbiri H, et al. Effect of cigarette smoking on coronary arteries and pattern and severity of coronary artery disease: A review. J Int Med Res 2021;49:03000605211059893; doi: 10.1177/03000605211059893 34855538 PMC8647272

[53] Al-Delaimy WK, Manson JE, Solomon CG, et al. Smoking and risk of coronary heart disease among women with type 2 diabetes mellitus. Arch Int Med 2002;162:273–279; doi: 10.1001/archinte.162.3.273 11822919

[54] Ngo AD, Chen JS, Figtree G, et al. Preterm birth and future risk of maternal cardiovascular disease–is the association independent of smoking during pregnancy? BMC Pregnan Childbirth 2015;15:1–11; doi: 10.1186/s12884-015-0571-7 PMC4491219 26141292

[55] Dewan N, Brabin B, Wood L, et al. The effects of smoking on birthweight-for-gestational-age curves in teenage and adult primigravidae. Public Health 2003;117:31–35; doi: 10.1016/s0033-3506(02)00003-3 12802902

[56] Delpisheh A, Kelly Y, Rizwan S, et al. Socio-economic status, smoking during pregnancy and birth outcomes: an analysis of cross-sectional community studies in Liverpool (1993–2001). J Child Health Care 2006;10:140–148; doi: 10.1177/1367493506062553 16707542

[57] Kucharska-Newton AM, Harald K, Rosamond WD, et al. Socioeconomic indicators and the risk of acute coronary heart disease events: Comparison of population-based data from the United States and Finland. Ann Epidemiol 2011;21:572–579; doi: 10.1016/j.annepidem.2011.04.006 21737046 PMC3132397

[58] Khoja A, Andraweera PH, Tavella R, et al. Pregnancy complications are associated with premature coronary artery disease: Linking three cohorts. J Womens Health 2023;32:1208–1218; doi: 10.1089/jwh.2023.0239. 37815882

[59] Aldridge E, Pathirana M, Wittwer M, et al. Effectiveness of a nurse practitioner-led cardiovascular prevention clinic at reduction of metabolic syndrome following maternal complications of pregnancy: A preliminary analysis. Diabetol Metab Syndr 2022;14:1–9; doi: 10.1186/s13098-022-00916-8 36203165 PMC9535230

